# Prevalence of Multiple Chronic Conditions Among US Adults: Estimates From the National Health Interview Survey, 2010

**DOI:** 10.5888/pcd10.120203

**Published:** 2013-04-25

**Authors:** Brian W. Ward, Jeannine S. Schiller

**Affiliations:** Author Affiliations: Jeannine S. Schiller, National Center for Health Statistics, Centers for Disease Control and Prevention, Hyattsville, Maryland.

## Abstract

Preventing and ameliorating chronic conditions has long been a priority in the United States; however, the increasing recognition that people often have multiple chronic conditions (MCC) has added a layer of complexity with which to contend. The objective of this study was to present the prevalence of MCC and the most common MCC dyads/triads by selected demographic characteristics. We used respondent-reported data from the 2010 National Health Interview Survey (NHIS) to study the US adult civilian noninstitutionalized population aged 18 years or older (n = 27,157). We categorized adults as having 0 to 1, 2 to 3, or 4 or more of the following chronic conditions: hypertension, coronary heart disease, stroke, diabetes, cancer, arthritis, hepatitis, weak or failing kidneys, chronic obstructive pulmonary disease, or current asthma. We then generated descriptive estimates and tested for significant differences. Twenty-six percent of adults have MCC; the prevalence of MCC has increased from 21.8% in 2001 to 26.0% in 2010. The prevalence of MCC significantly increased with age, was significantly higher among women than men and among non-Hispanic white and non-Hispanic black adults than Hispanic adults. The most common dyad identified was arthritis and hypertension, and the combination of arthritis, hypertension, and diabetes was the most common triad. The findings of this study contribute information to the field of MCC research. The NHIS can be used to identify population subgroups most likely to have MCC and potentially lead to clinical guidelines for people with more common MCC combinations.

## Introduction

Chronic conditions are an increasing concern in the United States, where they affect nearly half of the adult population and their prevalence has increased in recent years (1–3). These conditions result in numerous adverse health outcomes, increased health care needs, and subsequently higher medical costs (4–6). In the past, strategies have focused on preventing and ameliorating a single disease at a time; however, the large percentage of people with 2 or more conditions, or multiple chronic conditions (MCC), has added a layer of complexity to developing prevention and intervention strategies (7–10). As a result, the US Department of Health and Human Services (HHS) has developed a strategic framework to address MCC (11). Strategies of the framework include the stimulation of epidemiologic research to determine the most common MCC dyads and triads and to explain more clearly the differences in MCC and the opportunities for prevention and treatment among various sociodemographic groups (10,11).

Although numerous data sources are available to help meet these data-driven objectives, the National Health Interview Survey (NHIS) can be used to generate estimates of MCC that are representative of the noninstitutionalized, civilian adult population of the United States. The NHIS contains extensive sociodemographic and health data that can be studied with MCC. Furthermore, because it is conducted continuously, it can be used to monitor trends in MCC over time.

The main objective of this study was to use nationally representative data from the 2010 NHIS to examine the prevalence of MCC by select sociodemographic groups, and the prevalence of MCC dyads and triads among US adults. A secondary objective was to use earlier data from the NHIS to examine trends in MCC during 2001–2010.

## Analysis

### Data source

The NHIS is a multipurpose health survey that represents the US civilian, noninstitutionalized population (12,13). The NHIS is multistaged and is conducted continuously throughout each calendar year by using computer-assisted personal interviews. The survey has 3 main components: the Family Core, the Sample Adult Core, and the Sample Child Core. In the Family Core, an adult self-reports for himself or herself and as a proxy for the remainder of the family. From each family, 1 adult aged 18 years or older and 1 child (if the family includes a child) are randomly chosen for the Sample Adult Core and Sample Child Core questionnaires. The selected “sample adult” self-reports for the Sample Adult portion of the NHIS (unless a health condition requires a proxy respondent to answer for this adult) (12,13). All data for chronic conditions were taken from the Sample Adult Core, and data for sex, age, race/ethnicity, and health insurance coverage were taken from the Family Core. The 2010 NHIS Sample Adult Core contained 27,157 adults, had a conditional response rate (ie, the rate for those sample adults identified as eligible without taking into account household or family nonresponse) of 77.3%, and a final response rate of 60.8%. Descriptive estimates of select sociodemographic characteristics for the 2010 adult population are in Table 1. For the trend analysis using the NHIS for the years 2001 through 2010, the lowest Sample Adult Core sample size over the 10-year period was 21,781 (2008) and the highest was 33,326 (2001). The conditional response rate was lowest in 2008 (74.2%) and highest in 2003 (84.5%); the final response rate was lowest in 2008 (62.6%) and highest in 2002 (74.3%).

**Table 1:** Prevalence of Selected Sociodemographic Characteristics of US Adults, National Health Interview Survey, 2010

Variable	% (95% Confidence Interval)
**Sex**	
Men	48.3 (47.57–49.09)
Women	51.7 (50.91–52.43)
**Age, y**	
18–44	48.2 (47.32–49.08)
45–64	34.9 (34.19–35.70)
≥65	16.9 (16.26–17.47)
**Race/ethnicity**	
Non-Hispanic white	68.0 (67.13–68.91)
Non-Hispanic black/African American	11.6 (11.03–12.26)
Hispanic	14.0 (13.38–14.61)
Non-Hispanic Asian/Pacific Islander	4.7 (4.38–5.05)
Non-Hispanic American Indian/Alaska Native	0.5 (0.39–0.67)
Non-Hispanic other race	1.1 (0.99–1.33)
**Health insurance**	
Public	16.0 (15.38–16.59)
Private	62.9 (62.03–63.79)
Other	3.1 (2.87–3.44)
Uninsured	18.0 (17.34–18.60)

### Definitions

The HHS Interagency Workgroup on MCC and Office of the Assistant Secretary for Health have generated a standardized approach to defining chronic conditions in the United States (14), which was used as a basis to generate a measure of MCC. The 2010 NHIS included questions on 10 of the 20 chronic conditions captured by the Workgroup’s definition, including whether adults had ever been told by a doctor or other health professional that they had hypertension, coronary heart disease, stroke, diabetes, cancer, arthritis, hepatitis, or emphysema; had experienced weak or failing kidneys or chronic bronchitis during the past 12 months; or currently had asthma. Presence of emphysema or chronic bronchitis was combined in this analysis to form a single condition of chronic obstructive pulmonary disease (COPD). We counted the presence of each of these 10 conditions and combined them into 3 categories: 0 to 1 condition, 2 to 3 conditions, and 4 or more conditions. We also generated estimates for the 5 most common MCC dyad and triad combinations by sex and age group, and we estimated weighted prevalences for the 5 most common combinations. Within each combination, chronic conditions are listed alphabetically. These MCC dyad and triad combinations were not mutually exclusive; an adult could have more than 1 dyad or triad.

The NHIS questions on chronic conditions included in this article remained consistent for the 2001 through 2010 NHIS surveys, with 1 exception: in 2001 the NHIS asked sample adults if they had arthritis, with no reference to rheumatoid arthritis, gout, lupus, or fibromyalgia. From 2002 and onward, the question on arthritis included all 5 conditions (ie, arthritis, rheumatoid arthritis, gout, lupus, and fibromyalgia). The 10 conditions included in this HHS standardized approach (14) that were not measured by the NHIS for those years were congestive heart failure, cardiac arrhythmias, hyperlipidemia, autism spectrum disorder, dementia, depression, human immunodeficiency virus infection, osteoporosis, schizophrenia, and substance abuse disorders (a few of these conditions were measured by the NHIS in some years). The inability to capture these additional conditions, especially those related to mental health among the younger population (15), likely means percentages of MCC using the NHIS may be underestimates (16). Health insurance categories were based on a hierarchy of mutually exclusive categories (12,13), which included private coverage, public coverage (ie, Medicaid, Children’s Health Insurance Program, or Medicare), other coverage (ie, state-sponsored health plans, other government programs, or military health plans), and uninsured.

### Statistical analysis

To account for survey weights that allow for generalization to the US adult civilian noninstitutionalized population and the additional covariance resulting from the complex cluster sampling design used by the NHIS, we used SUDAAN version 10.0.1 (RTI International, Research Triangle Park, North Carolina) to generate all descriptive estimates and their corresponding confidence intervals. Two-tailed significance tests were used to test for significant differences in prevalence among population subgroups in 2010, and all differences noted in this article are significant (*P* < .05) unless otherwise noted. Estimates with a relative standard error greater than 30% were considered unreliable and were not discussed. Data from the 2001 through 2010 NHIS surveys were used to examine the trends of MCC by sex, age, and selected race/ethnicity subgroups with sufficient sample sizes to generate statistically reliable estimates. The JoinPoint Regression Program version 3.5.1 (National Cancer Institute, Washington, DC) was used to identify whether an increasing or decreasing trend was significant. This software also identified “joinpoints,” which are points in time where a change in trend occurs (17).

## Results

### Prevalence of MCC by sex and age

In 2010, 49.1% of civilian, noninstitutionalized US adults had no chronic conditions, and 24.8% had only 1 chronic condition. One-quarter of US adults had MCC (Table 2); 21.1% had 2 to 3 MCC and 4.9% had 4 or more MCC. For both sexes, older adults had a higher prevalence of MCC than younger adults. Among adults aged 18 to 44 years, men were less likely to have 2 to 3 MCC compared with women (*P* = .003). For adults aged 18 to 44 years and 45 to 64 years, men were less likely than women to have 4 or more MCC. In contrast, among adults 65 years or older, men were more likely than women to have 4 or more MCC.

**Table 2:** Prevalence of Chronic Conditions Among US Adults by Sex, Age, and Race/Ethnicity, National Health Interview Survey, 2010^a^

Sex, Age, and Race/Ethnicity	No. of Chronic Conditions		
	0–1, % (95% CI)	2–3, % (95% CI)	≥4, % (95% CI)
**Total**	74.0 (73.28–74.63)	21.1 (20.50–21.74)	4.9 (4.62–5.24)
**Men**			
**18–44 y**			
Total	93.3 (92.49–94.07)	6.3 (5.57–7.11)	0.4 (0.23–0.64)
Non-Hispanic white	93.6 (92.55–94.46)	6.1 (5.23–7.11)	0.3 (0.15–0.73)^b^
Non-Hispanic black/African American	91.2 (88.56–93.23)	8.0 (6.06–10.46)	0.8 (0.33–2.11)^b^
Hispanic	93.7 (91.76–95.21)	6.0 (4.53–7.93)	^—^ c
Non-Hispanic Asian/Pacific Islander	96.5 (93.51–98.13)	3.4 (1.76–6.36)^b^	^—^ c
Non-Hispanic American Indian/Alaska Native	86.7 (55.37–97.16)	^—^ c	^—^ d
Non-Hispanic other race	86.1 (73.41–93.26)	12.8 (5.89–25.64)^b^	^—^ c
**45–64 y**			
Total	67.2 (65.44–68.91)	28.1 (26.50–29.79)	4.7 (3.99–5.49)
Non-Hispanic white	66.3 (64.12–68.44)	28.4 (26.49–30.48)	5.2 (4.36–6.29)
Non-Hispanic black/African American	61.6 (56.57–66.30)	33.7 (29.13–38.69)	4.7 (3.24–6.79)
Hispanic	76.6 (72.26–80.50)	21.4 (17.61–25.65)	2.0 (1.19–3.38)
Non-Hispanic Asian/Pacific Islander	78.6 (71.56–84.27)	19.5 (14.08–26.45)	^—^ c
Non-Hispanic American Indian/Alaska Native	28.5 (10.28–58.20)^b^	66.8 (37.31–87.19)	^—^ c
Non-Hispanic other race	62.6 (44.99–77.41)	32.9 (18.26–51.92)	^—^ c
**≥65 y**			
Total	37.5 (35.17–39.85)	45.4 (42.92–47.88)	17.1 (15.31–19.13)
Non-Hispanic white	36.9 (34.24–39.62)	45.7 (42.91–48.60)	17.4 (15.29–19.68)
Non-Hispanic black/African American	32.3 (26.40–38.83)	48.6 (42.25–55.03)	19.1 (14.49–24.70)
Hispanic	47.5 (40.39–54.76)	40.1 (33.30–47.22)	12.4 (8.53–17.71)
Non-Hispanic Asian/Pacific Islander	39.4 (29.26–50.59)	45.1 (34.75–55.85)	15.5 (8.47–26.69)
Non-Hispanic American Indian/Alaska Native	^—^ d	^—^ d	100.0 (n/a)
Non-Hispanic other race	50.8 (29.75–71.58)	30.4 (13.70–54.70)^b^	^—^ c
**Women**			
**18–44 y**			
Total	91.1 (90.26–91.95)	8.0 (7.26–8.91)	0.8 (0.59–1.11)
Non-Hispanic white	91.0 (89.81–92.12)	8.2 (7.20–9.42)	0.7 (0.45–1.17)
Non-Hispanic black/African American	88.3 (86.00–90.20)	10.3 (8.48–12.45)	1.4 (0.87–2.38)
Hispanic	92.9 (91.19–94.23)	6.8 (5.42–8.42)	0.4 (0.19–0.73)^b^
Non-Hispanic Asian/Pacific Islander	97.1 (94.40–98.51)	2.8 (1.38–5.46)^b^	^—^ c
Non-Hispanic American Indian/Alaska Native	84.2 (63.87–94.17)	^—^ c	^—^ c
Non-Hispanic other race	83.2 (72.29–90.39)	12.9 (6.70–23.50)^b^	^—^ c
**45–64 y**			
Total	65.2 (63.64–66.73)	28.1 (26.65–29.56)	6.7 (5.97–7.54)
Non-Hispanic white	66.8 (64.87–68.62)	27.3 (25.55–29.06)	6.0 (5.08–6.98)
Non-Hispanic black/African American	52.0 (47.92–56.14)	36.8 (33.05–40.62)	11.2 (8.96–13.91)
Hispanic	65.9 (61.82–69.75)	26.7 (23.21–30.52)	7.4 (5.63–9.67)
Non-Hispanic Asian/Pacific Islander	79.9 (73.37–85.16)	18.6 (13.52–25.10)	1.5 (0.67–3.20)^b^
Non-Hispanic American Indian/Alaska Native	52.6 (31.78–75.52)	42.8 (23.61–64.50)	^—^ c
Non-Hispanic other race	48.5 (34.78–62.45)	27.7 (17.68–40.53)	23.8 (14.12–37.32)
**≥65 y**			
Total	38.2 (36.22–40.18)	47.4 (45.44–49.30)	14.5 (13.09–15.94)
Non-Hispanic white	38.2 (35.83–40.53)	47.4 (45.12–49.67)	14.5 (12.88–16.19)
Non-Hispanic black/African American	33.6 (28.98–38.45)	52.3 (47.29–57.18)	14.2 (11.13–17.93)
Hispanic	40.6 (34.82–46.75)	43.7 (38.03–49.50)	15.7 (11.48–21.04)
Non-Hispanic Asian/Pacific Islander	49.6 (40.80–58.43)	40.9 (32.76–49.54)	9.5 (5.33–16.42)
Non-Hispanic American Indian/Alaska Native	^—^ c	56.3 (25.52–82.89)	^—^ c
Non-Hispanic other race	^—^ c	47.7 (27.04–69.20)	32.7 (12.96–61.29)^b^

Abbreviations: CI, confidence interval; n/a, not applicable.

a Adults identifying as multiple races were included in the “other race” category.

b Relative standard error (RSE) >30% and ≤50% and should be used with caution as they do not meet National Center for Health Statistics standards of reliability and precision.

c RSE >50% are not shown.

d Estimates with a quantity of zero.

### Prevalence of MCC by sex, age, and race/ethnicity

Significant differences in the prevalence of MCC were found when the analysis was further stratified by race/ethnicity (Table 2). For example, among non-Hispanic white adults aged 18 to 44 years, men were less likely than women to have 2 to 3 MCC (*P* = .005), yet men aged 65 years or older were more likely to have 4 or more MCC than women 65 years or older. Among non-Hispanic black and Hispanic adults aged 45 to 64 years, women were more likely than men to have 4 or more MCC.

Differences in the prevalence of MCC were also found among specific racial/ethnic categories for certain sex/age groups (Table 2). Non-Hispanic American Indian/Alaska Native men aged 45 to 64 years were more likely to have 2 to 3 MCC compared with men 45 to 64 years in all other racial/ethnic groups. Among the same sex and age group (men 45 to 64 years), non-Hispanic white and non-Hispanic black men were more likely to have 4 or more MCC compared with Hispanic men; however, there was no significant difference in the prevalence of 4 or more MCC between non-Hispanic white and non-Hispanic black men. No significant racial/ethnic differences in the prevalence of 4 or more MCC were found among men aged 65 years or older.

For all 3 age groups, non-Hispanic black women had a higher prevalence of 2 to 3 MCC compared with Hispanic women (Table 2). Among those aged 45 to 64 years, non-Hispanic black women had a higher prevalence of 2 to 3 MCC relative to non-Hispanic white women. Non-Hispanic Asian/Pacific Islander women had a lower prevalence of 2 to 3 MCC than non-Hispanic American Indian/Alaska Native, non-Hispanic black, non-Hispanic white, and Hispanic women.

### Prevalence of MCC by sex, age, health insurance, and race/ethnicity

Table 3 further stratifies the estimates of MCC by health insurance coverage and shows that differences exist between different coverage statuses. Among men and women aged 18 to 44 years and 45 to 64 years, those with private coverage and those who were uninsured had a lower prevalence of 2 to 3 MCC than those with public coverage. Among women aged 45 to 64 years, the prevalence of 4 or more MCC was higher among those with public coverage than those with other coverage, without coverage, and private coverage. Prevalence of 4 or more MCC was significantly higher among men aged 45 to 64 years with public and other coverage than those with private coverage and without coverage.

**Table 3:** Prevalence of Chronic Conditions Among US Adults by Sex, Age, Health Insurance, and Race/Ethnicity, National Health Interview Survey, 2010^a^

Sex, Age, and Health Insurance, Race/Ethnicity	No. of Chronic Conditions		
	0–1, % (95% CI)	2–3, % (95% CI)	≥4, % (95% CI)
**Men**			
**18–44 y**			
Public, total	83.9 (79.44–87.53)	14.2 (10.78–18.47)	1.9 (0.89–4.06)^b^
Public, non-Hispanic white	81.9 (74.43–87.49)	16.2 (10.92–23.25)	^—^ c
Public, non-Hispanic black/African American	81.2 (71.56–88.17)	17.6 (10.78–27.34)	^—^ c
Public, Hispanic	89.7 (81.13–94.62)	8.1 (3.90–15.95)^b^	^—^ c
Private, total	94.0 (92.88–94.91)	5.9 (4.96–6.97)	^—^ c
Private, non-Hispanic white	94.6 (93.31–95.58)	5.4 (4.40–6.67)	^—^ d
Private, non-Hispanic black/African American	93.5 (90.04–95.87)	5.4 (3.40–8.32)	^—^ c
Private, Hispanic	91.6 (87.68–94.40)	8.2 (5.44–12.14)	^—^ c
Other, total	86.3 (77.85–91.83)	11.2 (6.25–19.16)	^—^ c
Other, non-Hispanic white	83.1 (69.69–91.33)	12.7 (5.88–25.45)^b^	^—^ c
Other, non-Hispanic black/African American	86.0 (60.53–96.12)	^—^ c	^—^ d
Other, Hispanic	92.0 (79.29–97.16)	^—^ c	^—^ d
Uninsured, total	94.7 (93.29–95.86)	4.9 (3.85–6.31)	^—^ c
Uninsured, non-Hispanic white	94.1 (91.62–95.91)	5.4 (3.70–7.69)	^—^ c
Uninsured, non-Hispanic black/African American	93.2 (89.11–95.87)	6.3 (3.75–10.41)	^—^ c
Uninsured, Hispanic	95.9 (93.68–97.33)	4.0 (2.58–6.20)	^—^ c
**45–64 y**			
Public, total	38.4 (32.85–44.23)	44.7 (39.10–50.39)	16.9 (13.27–21.38)
Public, non-Hispanic white	33.9 (26.36–42.35)	44.7 (37.04–52.54)	21.4 (15.97–28.17)
Public, non-Hispanic black/African American	41.3 (31.48–51.86)	47.8 (37.31–58.52)	10.9 (6.09–18.70)
Public, Hispanic	51.2 (37.37–64.92)	39.5 (26.52–54.22)	9.2 (4.54–17.84)^b^
Private, total	69.3 (67.14–71.35)	27.3 (25.37–29.40)	3.4 (2.60–4.35)
Private, non-Hispanic white	68.9 (66.43–71.34)	27.3 (25.07–29.73)	3.7 (2.79–4.95)
Private, non-Hispanic black/African American	62.1 (55.54–68.30)	34.5 (28.59–41.04)	3.3 (1.67–6.47)^b^
Private, Hispanic	74.9 (68.02–80.68)	23.9 (18.24–30.74)	^—^ c
Other, total	56.2 (48.84–63.28)	33.7 (27.13–40.96)	10.1 (6.92–14.54)
Other, non-Hispanic white	59.2 (49.57–68.17)	29.5 (21.52–38.94)	11.3 (7.12–17.53)
Other, non-Hispanic black/African American	42.3 (29.29–56.40)	48.7 (34.82–62.72)	9.1 (4.35–17.98)^b^
Other, Hispanic	59.3 (33.80–80.65)	38.5 (17.76–64.41)^b^	^—^ c
Uninsured, total	76.8 (72.88–80.35)	21.2 (17.70–25.14)	2.0 (1.15–3.44)
Uninsured, non-Hispanic white	71.4 (65.88–76.42)	25.9 (21.08–31.31)	2.7 (1.39–5.16)^b^
Uninsured, non-Hispanic black/African American	83.3 (75.61–88.95)	15.3 (9.99–22.81)	^—^ c
Uninsured, Hispanic	88.6 (82.71–92.67)	10.3 (6.42–16.22)	^—^ c
**≥65 y**			
Public, total	37.4 (34.17–40.77)	46.0 (42.47–49.49)	16.6 (14.13–19.46)
Public, non-Hispanic white	37.2 (33.15–41.39)	45.2 (40.88–49.58)	17.6 (14.49–21.27)
Public, non-Hispanic black/African American	29.0 (22.49–36.48)	55.0 (47.30–62.41)	16.0 (11.10–22.64)
Public, Hispanic	45.9 (37.71–54.35)	42.0 (33.84–50.66)	12.1 (8.10–17.59)
Private, total	36.6 (33.38–39.91)	45.5 (42.01–48.94)	18.0 (15.52–20.70)
Private, non-Hispanic white	36.2 (32.81–39.84)	46.3 (42.64–50.08)	17.4 (14.85–20.32)
Private, non-Hispanic black/African American	36.7 (25.22–49.85)	39.5 (28.98–51.01)	23.9 (14.56–36.58)
Private, Hispanic	46.0 (29.89–62.96)	36.0 (22.03–52.77)	18.0 (7.46–37.54)^b^
Other, total	56.3 (35.11–75.44)	28.2 (13.89–48.78)^b^	15.5 (6.19–33.88)^b^
Other, non-Hispanic white	68.0 (40.81–86.71)	21.5 (7.49–48.01)^b^	^—^ c
Other, non-Hispanic black/African American	^—^ c	^—^ c	37.0 (12.28–71.11)^b^
Other, Hispanic	^—^ d	100.0 (n/a)	^—^ d
Uninsured, total	67.2 (39.81–86.43)	30.7 (11.95–59.19)^b^	^—^ c
Uninsured, non-Hispanic white	48.1 (12.83–85.36)^b^	51.9 (14.64–87.17)^b^	^—^ d
Uninsured, non-Hispanic black/African American	^—^ c	^—^ c	^—^ c
Uninsured, Hispanic	82.8 (46.37–96.38)	^—^ c	^—^ d
**Women**			
**18–44 y**			
Public, total	84.5 (81.46–87.06)	12.9 (10.52–15.77)	2.6 (1.67–4.08)
Public, non-Hispanic white	79.4 (74.20–83.83)	17.2 (13.16–22.27)	3.3 (1.74–6.27)^b^
Public, non-Hispanic black/African American	84.7 (79.63–88.76)	11.9 (8.32–16.65)	3.4 (1.77–6.36)^b^
Public, Hispanic	92.5 (87.48–95.56)	7.0 (3.99–12.00)	^—^ c
Private, total	92.5 (91.42–93.51)	7.0 (6.05–8.12)	0.4 (0.25–0.80)^b^
Private, non-Hispanic white	92.9 (91.56–94.06)	6.7 (5.60–8.04)	0.4 (0.17–0.85)^b^
Private, non-Hispanic black/African American	89.3 (85.52–92.12)	10.0 (7.23–13.71)	^—^ c
Private, Hispanic	91.9 (88.95–94.09)	8.0 (5.78–10.91)	^—^ c
Other, total	90.3 (85.59–93.57)	8.9 (5.76–13.39)	^—^ c
Other, non-Hispanic white	89.9 (81.99–94.51)	8.8 (4.56–16.30)^b^	^—^ c
Other, non-Hispanic black/African American	89.2 (72.85–96.24)	^—^ c	^—^ d
Other, Hispanic	91.8 (85.22–95.58)	7.6 (3.97–14.20)^b^	^—^ c
Uninsured, total	91.7 (90.00–93.18)	7.6 (6.16–9.32)	0.7 (0.32–1.47)^b^
Uninsured, non-Hispanic white	90.9 (87.74–93.31)	8.6 (6.27–11.82)	^—^ c
Uninsured, non-Hispanic black/African American	89.7 (85.53–92.76)	9.4 (6.48–13.36)	^—^ c
Uninsured, Hispanic	94.2 (91.72–95.97)	5.3 (3.59–7.76)	0.5 (0.19–1.30)^b^
**45–64 y**			
Public, total	33.0 (28.80–37.58)	42.4 (38.17–46.73)	24.6 (20.90–28.64)
Public, non-Hispanic white	33.5 (27.06–40.60)	40.7 (34.32–47.34)	25.8 (20.77–31.65)
Public, non-Hispanic black/African American	24.5 (18.52–31.71)	53.1 (45.20–60.79)	22.4 (16.29–30.00)
Public, Hispanic	42.9 (32.46–53.93)	33.1 (24.09–43.55)	24.0 (16.04–34.40)
Private, total	68.9 (67.00–70.69)	26.7 (24.96–28.56)	4.4 (3.71–5.21)
Private, non-Hispanic white	70.0 (67.80–72.04)	26.1 (24.12–28.27)	3.9 (3.16–4.81)
Private, non-Hispanic black/African American	56.7 (51.08–62.23)	35.1 (30.22–40.42)	8.1 (5.63–11.55)
Private, Hispanic	68.9 (63.13–74.12)	25.5 (20.87–30.67)	5.6 (3.71–8.51)
Other, total	61.4 (53.20–68.95)	29.2 (22.62–36.83)	9.4 (5.63–15.31)
Other, non-Hispanic white	64.1 (53.02–73.78)	28.1 (19.43–38.80)	7.8 (3.47–16.73)^b^
Other, non-Hispanic black/African American	58.9 (41.83–74.06)	29.1 (16.85–45.49)	12.0 (5.18–25.27)^b^
Other, Hispanic	56.5 (37.64–73.71)	32.9 (18.07–52.24)	^—^ c
Uninsured, total	69.2 (65.15–73.06)	25.2 (21.66–29.17)	5.5 (3.84–7.89)
Uninsured, non-Hispanic white	67.1 (61.45–72.28)	26.4 (21.65–31.86)	6.5 (4.06–10.16)
Uninsured, non-Hispanic black/African American	68.8 (60.52–76.06)	24.1 (17.56–32.22)	7.0 (3.39–14.06)^b^
Uninsured, Hispanic	72.4 (64.16–79.31)	25.1 (18.53–33.05)	2.5 (0.96–6.50)^b^
**≥65 y**			
Public, total	36.9 (34.07–39.74)	47.2 (44.26–50.18)	15.9 (13.87–18.23)
Public, non-Hispanic white	37.4 (33.88–41.09)	46.0 (42.27–49.82)	16.6 (13.92–19.58)
Public, non-Hispanic black/African American	32.8 (26.91–39.19)	53.3 (46.84–59.66)	13.9 (10.58–18.14)
Public, Hispanic	36.9 (30.22–44.09)	48.4 (41.54–55.39)	14.7 (10.53–20.08)
Private, total	38.8 (36.01–41.65)	47.9 (45.15–50.72)	13.3 (11.52–15.27)
Private, non-Hispanic white	38.6 (35.57–41.76)	48.3 (45.25–51.38)	13.1 (11.19–15.21)
Private, non-Hispanic black/African American	33.6 (26.28–41.86)	52.5 (44.34–60.49)	13.9 (8.75–21.36)
Private, Hispanic	45.2 (31.56–59.62)	34.9 (23.78–48.00)	19.9 (10.35–34.76)^b^
Other, total	^—^ c	73.2 (37.60–92.52)	^—^ c
Other, non-Hispanic white	^—^ d	100.0 (n/a)	^—^ d
Other, non-Hispanic black/African American	^—^ c	^—^ d	^—^ c
Other, Hispanic	^—^ d	100.0 (n/a)	^—^ d
Uninsured, total	69.4 (47.49–85.00)	17.4 (6.66–38.32)^b^	^—^ c
Uninsured, non-Hispanic white	^—^ c	51.1 (15.43–85.71)^b^	^—^ c
Uninsured, non-Hispanic black/African American	60.3 (19.46–90.50)^b^	^—^ c	^—^ c
Uninsured, Hispanic	82.8 (48.96–96.04)	^—^ c	^—^ c

a Abbreviations: CI, confidence interval; n/a, not applicable.

b Relative standard error (RSE) >30% and ≤50% and should be used with caution as they do not meet National Center for Health Statistics standards of reliability and precision.

c RSE >50% are not shown.

d Estimates with a quantity of zero.

Analysis of differences in racial/ethnic groups revealed additional patterns in the prevalence of MCC by health insurance coverage. Non-Hispanic white men aged 45 to 64 years who had public coverage had a higher prevalence of 2 to 3 MCC than those who had private coverage (*P* < .001) and other coverage (Table 3). The prevalence of 4 or more MCC was higher among non-Hispanic white men aged 45 to 64 years with either public coverage or other coverage than those with private coverage (both *P* < .001). Among men aged 65 years or older, non-Hispanic black men with public coverage were more likely to have 2 to 3 MCC than non-Hispanic black men with private coverage.

Among non-Hispanic white women aged 45 to 64 years, those with public coverage had a higher prevalence of 2 to 3 MCC (*P* = .002) and 4 or more MCC (*P* < .001) than those with private coverage and those who were uninsured (Table 3). These same significant differences in the prevalence of 2 to 3 MCC were also found among non-Hispanic black women aged 45 to 64 years. In addition, non-Hispanic black women aged 45 to 64 years with public coverage also had a higher prevalence of 4 or more MCC than those with private health insurance coverage (*P* < .001). For Hispanic women aged 45 to 64 years, no significant differences were found in the prevalence of 2 to 3 MCC among health insurance coverage types, but those with public coverage had a much higher prevalence of 4 or more MCC than those with private coverage (*P* < .001). Among non-Hispanic white women aged 65 years or older, those with public coverage had a higher prevalence of 4 or more MCC than those with private coverage.

### Prevalence of MCC dyads and triads

To meet an additional objective of the HHS MCC framework — determination of the most common MCC dyads and triads (10,11) — we assessed the 5 most prevalent MCC dyad and triad combinations by sex and age group (Table 4). We list the individual chronic conditions within each dyad and triad alphabetically. For US men and women with at least 2 chronic conditions, for each age group, the MCC dyad with the highest prevalence was ever having had arthritis and ever having had hypertension. This MCC dyad was more prevalent among women aged 65 years or older compared with men 65 years or older (*P* < .001). The second most prevalent dyad for men in each age group was ever having had diabetes and ever having had hypertension. This dyad was also the second most prevalent for women aged 45 to 64 years and 65 years or older, where women 45 to 64 years were less likely to have diabetes/hypertension compared with men aged 45 to 64 years (*P* < .001). For women aged 18 to 44 years the second most prevalent dyad was ever having had arthritis and currently having asthma.

**Table 4:** Five Most Prevalent Chronic Condition Dyads for US Adults With 2 or More Chronic Conditions, by Sex and Age, National Health Interview Survey, 2010^a^

Sex, Age, and Dyad	% (95% Confidence Interval)
**Men**	
**18–44 y**	
Arthritis/hypertension	26.9 (22.01–32.43)
Diabetes/hypertension	21.1 (16.40–26.66)
Asthma/hypertension	18.6 (14.21–23.91)
COPD/hypertension	13.1 (9.12–18.36)
Arthritis/diabetes	9.2 (6.24–13.46)
**45–64 y**	
Arthritis/hypertension	46.9 (43.71–50.17)
Diabetes/hypertension	29.7 (27.02–32.50)
CHD/hypertension	16.4 (14.27–18.71)
Arthritis/diabetes	14.7 (12.70–17.05)
Cancer/hypertension	11.3 (9.50–13.43)
**≥65 y**	
Arthritis/hypertension	49.3 (46.29–52.32)
Diabetes/hypertension	29.5 (26.81–32.42)
Cancer/hypertension	27.6 (24.91–30.40)
CHD/hypertension	24.8 (22.05–27.84)
Arthritis/diabetes	21.2 (18.75–23.83)
**Women**	
**18–44 y**	
Arthritis/hypertension	24.6 (20.71–29.05)
Arthritis/asthma	20.5 (16.84–24.80)
Asthma/hypertension	19.5 (15.94–23.64)
Arthritis/COPD	16.7 (13.31–20.85)
Diabetes/hypertension	14.1 (11.12–17.74)
**45–64 y**	
Arthritis/hypertension	49.9 (47.24–52.55)
Diabetes/hypertension	23.6 (21.50–25.87)
Arthritis/diabetes	17.3 (15.38–19.37)
Asthma/hypertension	16.7 (14.72–18.89)
Arthritis/asthma	16.6 (14.79–18.63)
**≥65 y**	
Arthritis/hypertension	63.0 (60.46–65.51)
Diabetes/hypertension	25.4 (23.27–27.71)
Arthritis/diabetes	20.4 (18.39–22.50)
Cancer/hypertension	21.8 (19.78–24.02)
Arthritis/cancer	21.0 (19.05–23.07)

Abbreviations: COPD, chronic obstructive pulmonary disease; CHD, coronary heart disease.

a Within dyads, chronic conditions are listed in alphabetical order. Arthritis includes arthritis, rheumatoid arthritis, gout, lupus, and fibromyalgia.

Among US adults with at least 2 chronic conditions, the MCC dyad of ever having had arthritis and ever having had diabetes appeared across each of the various sex and age groups as 1 of the 5 most prevalent MCC dyads, with the exception of women aged 18 to 44 years (Table 4). Ever having had cancer and ever having had hypertension was 1 of the 5 most prevalent dyads among men aged 45 to 64 years and 65 years or older and women aged 65 years or older. Ever having had coronary heart disease and ever having had hypertension was 1 of the 5 most prevalent dyads among men aged 45 to 64 years and 65 years or older; however, this was not the case for women, regardless of age group.

As for the most prevalent MCC triads among US adults who had at least 3 chronic conditions (Table 5), for both men and women in most age groups the most prevalent triad was ever having had arthritis, ever having had diabetes, and ever having had hypertension. The one exception was for women 18 to 44 years, where the most common triad was ever having had arthritis, currently having asthma, and ever having had COPD. However, the arthritis/diabetes/hypertension triad was still one of the most prevalent among women aged 18 to 44 years with MCC.

**Table 5:** Five Most Prevalent Chronic Condition Triads for US Adults With 3 or More Chronic Conditions, by Sex and Age, National Health Interview Survey, 2010^a^

Sex, Age, and Triad	% (95 Confidence Interval)
**Men**	
**18–44 y**	
Arthritis/diabetes/hypertension	26.1 (16.70–38.45)
Asthma/diabetes/hypertension	15.5 (7.73–28.73)^b^
Arthritis/asthma/hypertension	14.6 (7.17–27.31)^b^
Arthritis/COPD/hypertension	12.2 (6.47–21.79)^b^
Arthritis/CHD/hypertension	7.3 (3.23–15.83)^b^
**45–64 y**	
Arthritis/diabetes/hypertension	28.3 (24.34–32.66)
Arthritis/CHD/hypertension	17.9 (14.52–21.86)
CHD/diabetes/hypertension	14.5 (11.37–18.22)
Arthritis/cancer/hypertension	11.2 (8.61–14.53)
Arthritis/asthma/hypertension	10.6 (8.03–13.91)
**≥65 y**	
Arthritis/diabetes/hypertension	28.2 (24.67–32.06)
Arthritis/cancer/hypertension	27.5 (23.97–31.31)
Arthritis/CHD/hypertension	27.2 (23.43–31.26)
CHD/diabetes/hypertension	17.8 (14.66–21.48)
Cancer/CHD/hypertension	14.6 (11.82–18.01)
**Women**	
**18–44 y**	
Arthritis/asthma/COPD	24.7 (17.68–33.50)
Arthritis/asthma/hypertension	21.3 (15.09–29.09)
Asthma/COPD/hypertension	19.8 (13.64–27.89)
Arthritis/COPD/hypertension	19.7 (13.82–27.32)
Arthritis/diabetes/hypertension	14.4 (9.65–21.03)
**45–64 y**	
Arthritis/diabetes/hypertension	30.5 (27.24–34.02)
Arthritis/asthma/hypertension	22.0 (19.00–25.35)
Arthritis/COPD/hypertension	18.4 (15.59–21.52)
Arthritis/cancer/hypertension	16.7 (13.80–20.09)
Arthritis/asthma/COPD	14.4 (12.08–17.16)
**≥65 y**	
Arthritis/diabetes/hypertension	32.6 (29.36–35.95)
Arthritis/cancer/hypertension	26.9 (23.95–30.13)
Arthritis/CHD/hypertension	19.3 (16.44–22.41)
Arthritis/COPD/hypertension	16.8 (14.19–19.84)
Arthritis/asthma/hypertension	16.5 (13.95–19.38)

Abbreviations: COPD, chronic obstructive pulmonary disease; CHD, coronary heart disease.

a Within triads, chronic conditions are listed in alphabetical order. Arthritis includes arthritis, rheumatoid arthritis, gout, lupus, or fibromyalgia.

b Relative standard error (RSE) >30% and ≤50% and should be used with caution as they do not meet National Center for Health Statistics standards of reliability and precision.

Another common MCC triad was ever having had arthritis, currently having asthma, and ever having had hypertension, which was 1 of the 5 most prevalent triads for each sex and age group with the exception of men aged 65 years or older (Table 5). Ever having had arthritis, ever having had cancer, and ever having had hypertension was prevalent among both men and women aged 45 to 64 years and 65 years or older; this prevalence was higher for women aged 45 to 64 years than for men aged 45 to 64 years.

### Trends in MCC

**Figure 1:**
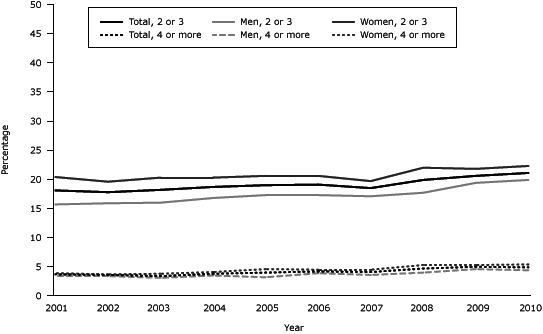
Prevalence of multiple chronic conditions among the total US adult population and separately, among US men and women, National Health Interview Survey for 2001 through 2010. **Table:** 

From 2001 through 2010, there was a slight (albeit significant) increasing trend among US adults for the prevalence of 2 to 3 MCC (*P* < .001) and 4 or more MCC (*P* < .001) (Figure 1). The same trend — slightly increasing and statistically significant — was also found when examining 2 to 3 MCC and 4 or more MCC separately for men and women.

Sex, No. of Conditions	2001	2002	2003	2004	2005	2006	2007	2008	2009	2010
Total, 2 or 3	18.1	17.8	18.2	18.7	19.0	19.1	18.5	19.9	20.6	21.1
Men, 2 or 3	15.7	15.9	16.0	16.8	17.3	17.3	17.1	17.7	19.4	19.9
Women, 2 or 3	20.4	19.6	20.3	20.3	20.6	20.6	19.7	22.0	21.8	22.3
Total, 4 or more	3.7	3.6	3.4	3.8	4.0	4.2	4.1	4.7	5.0	4.9
Men, 4 or more	3.4	3.4	3.1	3.5	3.2	3.9	3.6	4.0	4.6	4.4
Women, 4 or more	3.9	3.7	3.8	4.1	4.6	4.5	4.5	5.3	5.3	5.4

**Figure 2:**
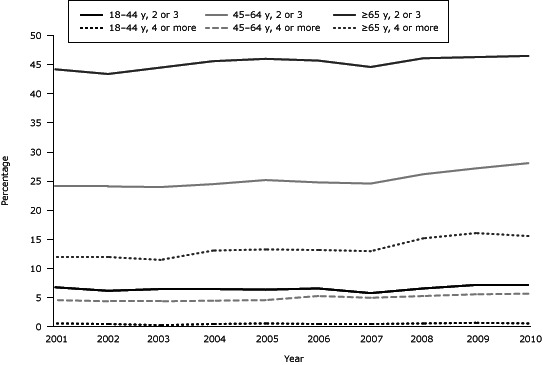
Prevalence of multiple chronic conditions among US adults aged 18 to 44 years, 45 to 64 years, and 65 years or older, National Health Interview Survey for 2001 through 2010. **Table:** 

Examination of the 2001–2010 NHIS data for MCC by age showed no significant increase in the prevalence of 2 to 3 MCC or 4 or more MCC for adults aged 18 to 44 years (Figure 2). For adults 65 years or older, there were slight significant increases from 2001 to 2010 in the prevalence of both 2 to 3 MCC (*P* = .005) and 4 or more MCC (*P* < .001). For adults aged 45 to 64 years with 2 to 3 MCC, the increasing trend was significant (*P* = .005) during 2007 through 2010. A significant increase in the prevalence of 4 or more MCC from 2001 through 2010 was found among adults aged 45 to 64 years.

Age (y), No. of Conditions	2001	2002	2003	2004	2005	2006	2007	2008	2009	2010
18–44, 2 or 3	6.8	6.2	6.5	6.5	6.4	6.6	5.8	6.6	7.2	7.2
45–64, 2 or 3	24.1	24.1	24.0	24.5	25.2	24.8	24.6	26.2	27.2	28.1
≥65, 2 or 3	44.2	43.4	44.5	45.6	46.0	45.7	44.6	46.1	46.3	46.5
18–44, 4 or more	0.6	0.5	0.3	0.5	0.6	0.5	0.5	0.6	0.7	0.6
45–64, 4 or more	4.6	4.4	4.4	4.5	4.6	5.3	5.0	5.3	5.6	5.7
≥65, 4 or more	12.0	12.0	11.5	13.1	13.3	13.2	13.0	15.2	16.1	15.6

**Figure 3:**
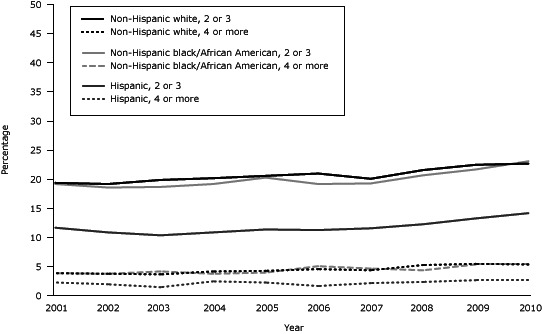
Prevalence of multiple chronic conditions among non-Hispanic white, non-Hispanic black, and Hispanic adults in the United States, National Health Interview Survey for 2001 through 2010. **Table:** 

For the prevalence of MCC from 2001 through 2010 by race/ethnicity (Figure 3), a slight increase in 2 to 3 MCC was found among non-Hispanic white adults (*P* < .001). However, for non-Hispanic black adults (*P* = .04) and Hispanic adults (*P* = .03), the increasing trend was significant only during 2007 through 2010. Among non-Hispanic white and non-Hispanic black adults there was a slight, significant increase in the prevalence of 4 or more MCC over time. This trend in the prevalence of 4 or more MCC was not significant for Hispanic adults.

Race, No. of Conditions	2001	2002	2003	2004	2005	2006	2007	2008	2009	2010
Non-Hispanic white, 2 or 3	19.4	19.2	19.9	20.2	20.6	21.0	20.1	21.6	22.5	22.7
Non-Hispanic black/African American, 2 or 3	19.2	18.6	18.7	19.2	20.3	19.2	19.3	20.7	21.7	23.1
Hispanic, 2 or 3	11.7	10.9	10.4	10.9	11.4	11.3	11.6	12.3	13.3	14.2
Non-Hispanic white, 4 or more	3.9	3.8	3.7	4.2	4.3	4.6	4.4	5.3	5.5	5.4
Non-Hispanic black/African American, 4 or more	3.9	3.8	4.2	3.8	4.0	5.1	4.7	4.4	5.4	5.4
Hispanic, 4 or more	2.3	2.0	1.5	2.5	2.3	1.7	2.2	2.4	2.7	2.7

## Summary

The main objective of our study was to use the NHIS to examine the prevalence of MCC by select sociodemographic groups and the prevalence of MCC dyads and triads. The results showed that more than one-quarter of US adults have MCC. Among certain subgroups (such as women and older adults), the prevalence of MCC was generally higher, and for others (Hispanic adults and those with private insurance) the prevalence was generally lower. Not surprisingly (18), the prevalence rates of our study vary from those of others because of differing populations of interest and the specific definition of MCC used. However, some of the general patterns found in our study, such as higher prevalence among older adults, have also been found in past research (8,19). The arthritis/hypertension dyad and the arthritis/diabetes/hypertension triad were 2 of the most prevalent MCC combinations, differing from the most common MCC combinations found by other studies examining adults aged 65 or older (20,21). Our trend analyses showed significant increases in MCC for all adults since 2001.

Although MCC prevalences presented in this study are generalizable to the US adult noninstitutionalized civilian population, use of the NHIS has limitations. Only 10 conditions detailed in the HHS Interagency Workgroup definition (14) were able to be captured, leaving certain conditions unaccounted for (15,16). Of the conditions captured, their measurement using NHIS could potentially be further debated (eg, including all cancers as opposed to only noncurable cancers). The NHIS also captured only conditions that were confirmed by a doctor or health professional, potentially leading to the underreporting of conditions that remain undiagnosed or were not recalled by the respondent during the NHIS interview. Finally, this research was exploratory in nature and used multiple comparisons, which could increase the likelihood of type I error.

In spite of these limitations, examining the prevalence of MCC among subgroups of adults allows for the identification of MCC patterns in the US adult population. Our research serves as a platform from which additional research using the NHIS can build. It would be beneficial for future studies to seek to explain why differences in the prevalence of MCC among subgroups exist. This might entail examining topics such as how different health insurance types influence service use and the likelihood of being diagnosed with a chronic condition, how educational attainment may affect MCC, or what behavioral risk factors are most common among adults with MCC.

Our study shows that the increasing trend in the prevalence of MCC among US adults is a cause for concern, and the NHIS can be a useful data source for identifying patterns of MCC at the national level and assessing which population subgroups are most likely to have MCC. This information can be useful in helping clinicians develop prevention strategies tailored to population subgroups with greater prevalence of MCC and subgroups that are most at risk for complications resulting from specific dyads and triads, consequently reducing health care costs among these subgroups.
